# Xanthoceraside Could Ameliorate Alzheimer’s Disease Symptoms of Rats by Affecting the Gut Microbiota Composition and Modulating the Endogenous Metabolite Levels

**DOI:** 10.3389/fphar.2019.01035

**Published:** 2019-09-13

**Authors:** Hongxu Zhou, Jingjie Tai, Haiyan Xu, Xiumei Lu, Dali Meng

**Affiliations:** ^1^School of Traditional Chinese Materia Medica, Shenyang Pharmaceutical University, Shenyang, China; ^2^School of Pharmacy, Shenyang Pharmaceutical University, Shenyang, China

**Keywords:** Alzheimer’s disease, xanthoceraside, gut microbiota, 16S rRNA gene sequencing, metabolomics

## Abstract

Xanthoceraside (XAN) is a natural-derived compound with anti-Alzheimer activity from the husks of *Xanthoceras sorbifolia*. Although its therapeutic effect had been confirmed in previous studies, the mechanism was still unclear due to its poor solubility and low permeability. In this study, the pharmacological effect of XAN on Alzheimer’s disease (AD) was confirmed by behavior experiments and H&E staining observation. Fecal microbiota transplantation (FMT) experiment also replicated the therapeutic effects, which indicates the potential targets of XAN on gut microbiota. The sequencing of 16S rRNA genes in fecal samples demonstrated that XAN reversed gut microbiota dysbiosis in AD animals. XAN could change the relative abundances of several phyla and genus of bacterial, particularly the ratio of *Firmicutes/Bacteroidetes*. Among them, *Clostridium IV*, *Desulfovibrio*, *Corynebacterium*, and *Enterorhabdus* had been reported to be involved in the pathologic developments of AD and other central nervous system disease. In metabolomics study, a series of host endogenous metabolites were detected, including amino acids, lysophosphatidylcholine, dihydrosphingosine, phytosphingosine, inosine, and hypoxanthine, which were all closely associated with the development of AD. Combined with the Spearman’s correlation analysis, it was confirmed that the increases of five bacterial strains and decreases of six bacterial strains were closely correlated with the increases of nine host metabolites and the decreases of another five host metabolites. Therefore, XAN can modulate the structure of gut microbiota in AD rats; the changes of gut microbiota were significantly correlated with endogenous metabolites, and symptom of AD was ultimately alleviated. Our findings suggest that XAN may be a potential therapeutic drug for AD, and the gut microbiota may be potential targeting territory of XAN *via* microbiome–gut–brain pathway.

## Introduction

Alzheimer’s disease (AD) is a neurodegenerative disorder characterized by a series of symptoms including progressive cognitive deterioration, memory loss, and progressive functional dependence, which eventually leading to death. The most convincing hypothesis of AD pathophysiological mechanism is the accumulation of extracellular amyloid-β (Aβ) and formation of neurofibrillary hyperphosphorylated tau (*p*-tau) tangle (Nisbet et al., 2015). However, lots of candidates, such as solanezumab, avagacestat, and verubecestat that are explored based on this hypothesis, have all failed in the III clinical trials because of their low efficiencies, indicating the difficulty and impossibility of mono-brain-target design during anti-AD drug studies. Hence, this theory only presents partial interpretation instead of a big picture on the treatment of AD.

Recently, accumulating clinical and experimental evidences suggest that gut microbiota (GM) play an essential role in central nervous system (CNS) diseases, including Parkinson diseases (PD), autism spectrum disorders (ASD), and AD (Hsiao et al., 2013; Kumar et al., 2016; Sampson et al., 2016; Zhang et al., 2017). More inspirationally, recent research indicates an important physiologic role of the Aβ protein as an antimicrobial agent to eliminate bacterial infection in the brain (Kumar et al., 2016). GM might be involved in the development of Aβ aggregation and change the level of SCFAs in APPPS1 transgenic mice (Harach et al., 2017; Zhang et al., 2017); what’s more, its dysbiosis aggravates the progression of AD in *drosophila* (Wu et al., 2017). Consequently, it could be proposed that there’s a close connection between GM and AD through microbiota–gut–brain axis, and GM could be served as a potential new target for therapeutic intervention in AD.

Xanthoceraside (XAN, **Figure S1A**) is a triterpenoid from the husks of *Xanthoceras sorbifolia* Bunge. In our previous studies, it was found that XAN could exert anti-AD effect through a quite different way from donepezil, a traditional AChE inhibitor. It could up-regulate BDNF expression, lower down the aggregation of Aβ, as well as be against with oxidative stress and synaptic damage in several AD animal models without affecting AChE activities (Chi et al., 2013; Liu et al., 2013; Qi et al., 2013; Jin et al., 2014; Li et al., 2016; Ji et al., 2017). But these studies focused only on the ultimate targets in brain without further insight on its integral bioactivity. Actually, XAN could hardly be absorbed into the blood and blood–brain barrier (BBB) due to its poor solubility and low permeability (Meng et al., 2016). Therefore, XAN might exhibit its bioactivity in gut tract by affecting the GM structures instead of adsorbing into blood. So, it’s of great importance to evaluate its effects on the intestinal microbial metabolism, which would change the level of endogenous substances in the body and improve the pathology of AD through microbiota–gut–brain axis.

The 16S rRNA gene sequencing approach is a preferable method which could detect fastidiously or non-cultivable organisms through amplification and determinate the sequence of conserved genes or culture-independent profiling. Metabolomics has emerged as a powerful approach in systems biology, focusing on a holistic investigation of the small molecular response of living systems to external stimuli, as well as providing diagnostic information and presenting mechanistic insight into biochemical effects of drugs (Zhao et al., 2008). The combined technology of 16S rRNA gene sequencing and metabolomics will provide a new sight to understand the relationship between GM and CNS diseases as early reported (Yu et al., 2017). In this study, AD rat model induced by the Aβ_1–42_ was established to explore the GM variation after XAN administration, and the underlying metabolic mechanisms were revealed. A novel strategy of integrated 16S rRNA gene sequencing with UPLC-/MS-based metabolomics was applied to analyze the relationship among GM, metabolites, and AD, which would finally contribute to the recognization of the integral activity of XAN on AD.

## Materials and Methods

### Materials

The husks of *X. sorbifolia* were collected from Chifeng, the Nei Mongol Autonomous Region, China (lat. 41°17′10″ N, long. 116°21′07″E; altitude 300 m a.s.l.), in a dry season Oct 2011. A voucher specimen (NO.WGG-1110) was deposited in the Department of Natural Products Chemistry, Shenyang Pharmaceutical University, China. XAN was separated from 10.0 kg of husks according to our previous study (Meng et al., 2016), which was dissolved in distilled water (1% DMSO) for oral administration by gavage. The HPLC system (Agilent HPLC-1260 Infinity, Santa Clara, California, USA; Diamonsil C18 column, 250 mm × 4.6 mm, 5 µm, Beijing DIMAGIT Science & Technology Co., Ltd., Beijing, China; UV detector 210 nm) revealed that the purity of XAN was higher than 99.0% as shown in **Figure S2** (**Supplementary Files**).

Aβ protein fragment 1–42 (Aβ_1–42_) was purchased from Sigma–Aldrich (St. Louis, MO, USA). Donepezil hydrochloride tablets was purchased from Eisai China Inc. (Tokyo, Japanese). Acetonitrile and formic acid of HPLC grade were purchased from Tedia (Fairfield, OH, USA) and Dikma Corp. (Richmond Hill, NY, USA).

### Animals and Experimental Design

Male SD rats (200–250 g, license no. SCXK [Liao] 2015-0001) were obtained from Changsheng Biotechnology Co., Ltd. (China). The rats were housed in ICV cages (five rats/cage) and kept in a regulated environment (23 ± 2°C, 50 ± 5% humidity and 12h-/12-h light/dark cycle) with food and water freely available. In subsequence experiments, all rats were housed individual cage under SPF and controlled environmental conditions. All the animal studies were performed in strict accordance with the guidelines of Chinese Society of Laboratory Animal Sciences to minimize the suffering of the animals throughout the study. Behavioral experiments were carried out in a sound-attenuated and air-regulated experimental room, in which rats were habituated beforehand for at least 1 h.

### Groups and Experimental Design

Aβ_1–42_ was dissolved in sterile physiological saline and aggregated by incubating with a concentration of 1 mg/ml at 37°C for 5 days before the injection. Briefly, 8-week-old rats were divided into eight groups randomly, including three XAN-treated groups (XAN-L 0.056, XAN-M 0.112, or XAN-H 0.224 mg/kg), positive drug group (donepezil, 0.6 mg/kg), model group and sham operation group, AXF group (AD animals with XAN-H group fecal), and ASF group (AD animals with sham operation group fecal). The rats received a left lateral ventricle injection of 5 µl aggregated Aβ_1–42_ by the stereotaxic apparatus (AP, +0.5 mm; ML, −1.1 mm; DV, −3.0 mm relative to the bregma) (Guo et al., 2011; Frozza et al., 2013; Stepanichev et al., 2014; Mao et al., 2015; Gupta et al., 2018; Kumar et al., 2019; Schimidt et al., 2019) for model and drug administration groups. The sham operation group was injected with equivalent volumes of physiological saline. After Aβ_1–42_ injection, the rats were intragastrical administrated with XAN or donepezil 1 h before the behavioral tasks once daily (**Figure S1B**) till the last day of the study. Equivalent distilled water was given intragastrically to the model and sham groups.

### Gut Microbiota Transplantation

The fecal microbiota transplantation (FMT) was performed based on an established protocol (Borody et al., 2013; Chang et al., 2015; Zhao et al., 2018). Fecal samples were collected from XAN-H groups and sham operation group at the17–18^th^ day. FMT experiment started from the third day after AD model induction (n = 6 for each transplant group, 8-week-old rats). The Y-maze test was performed at the 12^th^ day of FMT. The Morris water maze test (MWM) was performed at the 14–19^th^ day (**Figure S1C**). Briefly, each fecal sample (1 g) was diluted in 10 ml sterile 0.9% sodium chloride solution (w/v). The fecal material was suspended by thorough vortexing (3 min) and settled by gravity for 5 min. The suspension was filtered through a 1-mm sieve. The AXF and ASF group rats were intragastrical administrated with fecal suspension (1 g/kg body weight) at the 3–19^th^ day. The food and environmental conditions of AXF and ASF group rats were consistent of other group rats.

### Behavior Study

#### Y-Maze Test

The Y-maze test was performed at the ninth day of drug administration. The device consists of three wooden arms (each arm was 40-cm length, 5-cm width, and 12-cm height) which were 120° so as to allow the rats to freely enter into the three arms. Rats were placed at one arm end and allowed to explore freely for 8 min, and the sequences of arm entries were then recorded. Alternation was defined as continuous entering into three different arms. The alternation behavior (%) was calculated according to the following formula: (numbers of successful alternation/[total number of arm entries—2]) ×100%.

#### Morris Water Maze Test

Spatial learning and memory ability of the animals were tested by MWM at the 11–16^th^ day. The apparatus is composed of a circular water tank (140 cm in diameter and 44 cm in height) and a removable platform at the fourth quadrant. The temperature of the water was controlled below 22°C, and the depth was 30 cm. The platform (10 cm in diameter) was placed 1-cm underwater. Each rat was trained twice a day for five consecutive days with an interval of 3 h. Rats were trained to swim escaping onto the platform from different positions in the first quadrant. The rats who failed to find the platform within 90 s were guided to the platform and stayed for 10 s. After the orientation navigation test on the sixth day, the spatial exploration test was conducted while the platform was removed from the pool. The time spent in the target quadrant was measured. All the data were recorded automatically with computer software (designed by Institute of Materia Medica, Chinese Academy of Medical Science).

#### Sample Collection and Preparation

The plasma and brain of rats were collected after the end of behavior tests. Each 200 µl aliquot of plasma sample was mixed with 400 µl of acetonitrile and vortexed to precipitate the proteins. Brain samples were homogenized with water (10 ml/g) in ice bath. Three times the amount of methanol was added into brain homogenate twice. After centrifuged at 12,000 rpm for 10 min at 4°C, the supernatants were transferred and evaporated to dryness at 35°C under a gentle stream of nitrogen. The dried residues were then reconstituted in 100 µl of 80% acetonitrile solution, and 5 µl aliquot of supernatant was injected for UPLC/MS analysis.

Fecal pellets were obtained from each rat with the metabolic cage within 24 h after the end of behavioral tests. They were placed in sterile conical tubes and immediately frozen at −80°C for microbial community analysis.

#### H&E Staining

The rats were deeply anaesthetized and transcardially perfused with heparinized saline followed by 4% buffered formalin solution (pH 7.4). The brains were removed and embedded in paraffin, and 5-µm thick coronal sections were prepared for subsequent staining. Mounted sections were rinsed with distilled water and stained with alum hematoxylin. After rinsing in distilled water, sections were stained with eosin for 2 min and dehydrated for mounting.

#### UPLC/MS Analysis

The UPLC/MS analysis was carried out with a Waters ACQUITY Ultra Performance Liquid Chromatography (UPLC) System (Waters Corp., Milford, USA) coupled with a Micromass Quattro Micro API Mass Spectrometer (Waters Corp., Milford, MA, USA). The UPLC column used was a 100 mm × 2.1 mm, 1.7 µm, C_18_ column (Waters Corp., Milford, MA, USA). The column temperature was set at 40°C. The gradient mobile phase was a mixture of 0.1% formic acid in water (A) and 0.1% formic acid in acetonitrile (B), which was pumped at the ﬂow rate of 0.2 ml/min without split. All the samples were kept at 4°C during the analysis. The elution gradient program for plasma begins with 100% A and maintained for 0.5 min then decreased linearly to 5% A within 20 min and held for 1 min, followed by re-equilibration at 100% for additional 3 min. For the brain tissue, the solvent composition was 95–89% A from 0 to 2 min and 89–83% A from 2 to 2.5 min, then 83–77% A from 5 to 6 min, followed by a ramp of curve 77–58% A from 6 to 25 min, and re-equilibration at 95% A for 5 min. An electrospray ionization source (ESI) interface was used and was set in both positive and negative modes so as to monitor as many ions as possible. The following parameters were employed: source temperature of 120°C and desolvation temperature of 350°C, capillary voltage of 3.0 kV and 2.8 kV for positive and negative ionization modes, respectively; cone voltage for the plasma and the brain was 35 and 30 V, respectively. Nitrogen was used as the desolvation and cone gas with the flow rate of 400 and 30 L/h, respectively. MS data were collected in the full scan mode from m/z 100 to 1,000 amu over 0–24 min. Potential biomarkers were analyzed by UPLC/MS/MS. Argon was employed as the collision gas, and the collision energy was altered between 5 and 25 eV. NaCsI was used for mass correction before the study. The mass spectrometric data were collected in centroid mode.

#### Method Validation

The applied method was validated prior to the analysis of the experimental samples according to our previous study (Lu et al., 2011), including the precision of injection, method repeatability, post-preparative stability, and stability of freeze–thaw process. In addition, one quality control sample was inserted every 10 real samples during the whole sample analysis to further monitor the stability of the analysis.

#### Data Analysis

All UPLC/MS data acquired were processed using the MarkerLynx within MassLynx software version 4.1 (Waters Corp., Milford, USA) for peak detection and alignment. All data were normalized to the summed total ion intensity per chromatogram. Multivariate data analysis was performed with SIMCA-P 11.5 software package (Umetrics, Umea, Sweden) to select distinct variables. ANOVA was performed in succession to reveal the statistical differences for the variables among groups. The potential biomarkers with significant difference were identified according to their mass spectra and MS/MS spectra compared with those in databases, such as HMDB (http://www.hmdb.ca/), KEGG (http://www.genome.jp/kegg), METLIN (http://metlin.scripps.edu), and MassBank (http://www.massbank.jp). Some metabolites were further confirmed by comparing their retention times, mass spectra, and MS/MS spectra with authentic chemicals.

All data collected in behavior study (Y-maze test and MWM test) were expressed as mean ± SD. Statistics significance was assessed by ANOVA test by SPSS v 21.0 (IBM, Chicago, IL, USA). Confidence level was set at 95% to determine the significance of difference (*p* < 0.05).

#### 16S rRNA Gene Sequence Analysis

Total DNA of fecal samples was extracted using E.Z.N.A.^®^ Soil DNA Kit (OMEGA). The V3–V4 region of the 16S rRNA gene was selected for the subsequent pyrosequencing. PCR amplification was carried out using bacterial primers 341F (5’-CCTACACGACGCTCTTCCGATCTN-3’) and 805R (5’-GACTGGAGTTCCTTGGCACCCGAGAATTCCA-3’). The PCR products were carried out using QIIME software package and sequenced using on an Illumina HiSeq platform. The filtered sequences were clustered into operational taxonomic units (OTUs) according to representative sequences using USEARCH (Hartmann et al., 2012) and classified against the Greengenes Database (Desantis et al., 2006) with a threshold of 97% sequence similarity. OTUs were assigned to tree-based alpha- and beta diversity analyses.

## Results

### Behavior Experiment Results

#### The Y-Maze Test

**Figure 1:**
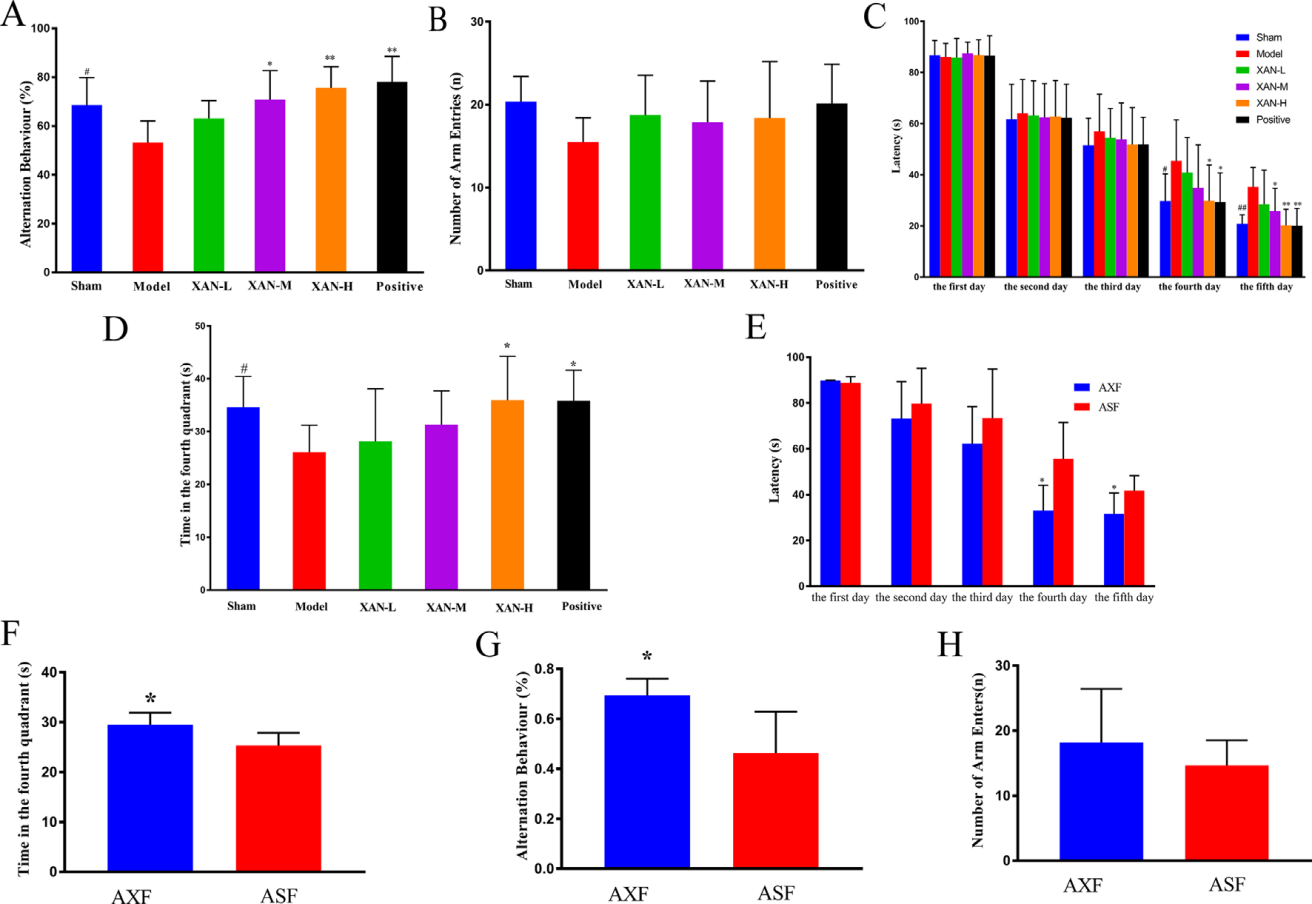
Effects of XAN on the impairment of spontaneous alteration behavior induced by Aβ_1–42_ in the Y-maze test and the water maze test. All results expressed as mean ± SD (n = 8, except in positive drug group, n = 6). **(A)** Alternation behavior. **(B)** Total number of arm entries. **(C)** Escape latency in orientation navigation test. **(D)** Time in fourth quadrant in spatial exploration test. **(E)** AXF and ASF rats’ escape latency in orientation navigation test; **(F)** AXF and ASF rats’ time in fourth quadrant in spatial exploration test. **(G)** AXF and ASF rats’ alternation behavior; **(H)** AXF and ASF rats’ total number of arm entries. AXF, AD animals with XAN-H group fecal; ASF, AD animals with sham operation group fecal (n = 6/group, ^#^ *p* <0.05 *vs.* sham group; *p <0.05, **p <0.01 *vs.* model group; the results are expressed as mean ± SD).

Y-maze test was performed to evaluate the effects of XAN on the memory impairment induced by Aβ_1–42_. Model rats showed significantly reduced spontaneous alternation behavior compared with sham group rats (**Figure 1A**). XAN (0.112 or 0.224 mg/kg) and donepezil significantly attenuated the impairment of spontaneous alternation behavior in model group rats (**Figure 1A**), in which the effect of XAN was in a dose-dependent manner. No significant differences in the total number of arm entries were found among groups, indicating that Aβ_1–42_, XAN, and donepezil did not influence spontaneous locomotor activity in rats (**Figure 1B**).

#### Morris Water Maze Test

Water maze test was performed to investigate the effects of XAN on the spatial memory ability of the rats. During the training period, escape latencies of all groups were shortened gradually with the increasing of training time. From day 3 of the water maze test, model group rats spent longer time to find the safe platform than sham group rats (**Figure 1C**), which demonstrated that the model was established successfully. Meanwhile, XAN (0.112 or 0.224 mg/kg) took a significant effect on the last two training days compared with the model group (*p* < 0.05), and the similar effects were found in donepezil treatment group. In the spatial exploration test, the total time spent in the fourth quadrant of model group rats were significantly reduced compared with sham operation group rats (**Figure 1D**), while rats in the treatment groups (XAN 0.224 mg/kg or donepezil 0.6 mg/kg) spent more time and traveled longer length to search in the fourth quadrant compared with the Aβ-injected mice, indicating a better spatial memory ability. The effect of XAN was in a dose-dependent manner, and there were statistic significances at 0.112 and 0.224 mg/kg XAN–treated group and 0.6 mg/kg donepezil–treated group (*p* < 0.05).

#### Gut Microbiota Transplantation

In Y-maze test, the AXF group rats showed notably increased spontaneous alternation behavior compared with ASF group rats (**Figure 1G**). There were no significant differences in the total numbers of arm entries between two groups (**Figure 1H**). In water maze test, the escape latencies of AXF group rats were significantly reduced compared with ASF group rats from fourth day (**Figure 1E**). In the spatial exploration test, the total time spent in the fourth quadrant of AXF group rats were significantly increased compared with ASF group rats (**Figure 1F**).

#### H&E Staining

**Figure 2:**
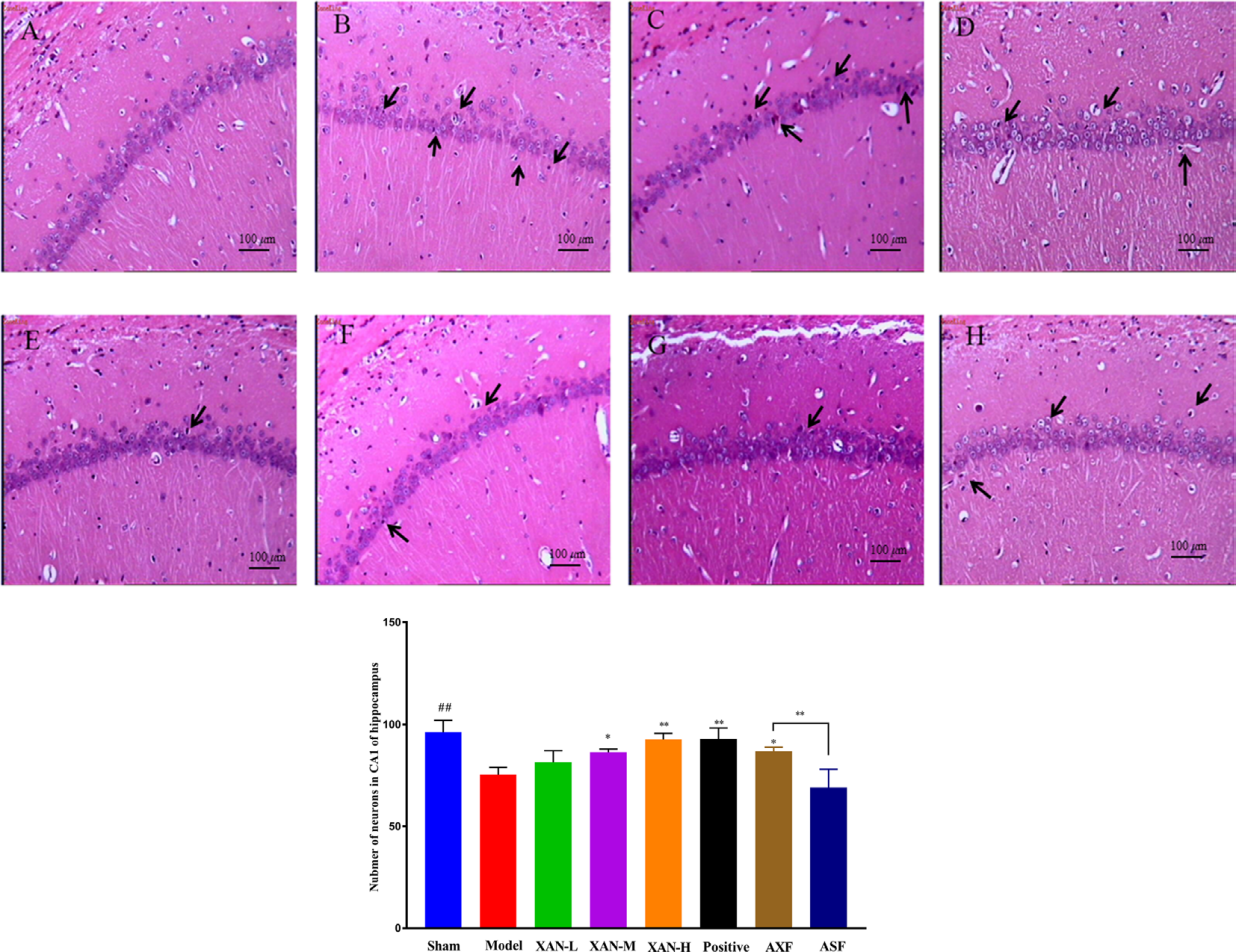
H&E staining. **(A)** Sham operation group. **(B)** AD model group. **(C)** XAN low-dose group. **(D)** XAN middle-dose group. **(E)** XAN high-dose group. **(F)** positive drug group. **(G)** AXF group, AD animals with XAN-H group fecal). **(H)** ASF group, AD animals with sham operation group fecal (100 × magnification, scale bar: 100 µm, n = 6/group, ^##^ *P* < 0.01 *vs*. sham group; *p < 0.05, **p < 0.01 *vs*. model group; the results are expressed as mean ± SD).

The layer of CA1 pyramidal cells in AD model group became thinner with pyknotic nuclei (more hippocampal neuronal loss) than sham operation group in H&E staining (**Figure 2**). Meanwhile, XAN treatment groups had significantly fewer hippocampal neuronal loss than the model group, and the similar effects were found in donepezil treatment group and AXF group (**Figure 2**).

#### UPLC/MS Method Validation

Six extracted ions from different plasma chromatogram regions were selected as followed: 4.4_188.0, 8.5_218.2, 11.5_355.1, 14.7_544.1, 19.9_552.2, and 22.3_226.8 with retention time covering the whole analytical time. While for brain tissue method validation, those extracted ions were 1.0_132.0, 1.4_269.1, 9.5_387.1, 12.1_318.0, 13.2_526.0, and 15.6_342.0.

The RSDs of retention times were less than 1.2%, and the RSDs or REs of MS responses for the typical peaks were less than 19%. All the results indicated the method was robust with good repeatability and stability, which could be used in analyzing large numbers of metabonomic samples.

#### Metabonomic Profiling

**Figure 3:**
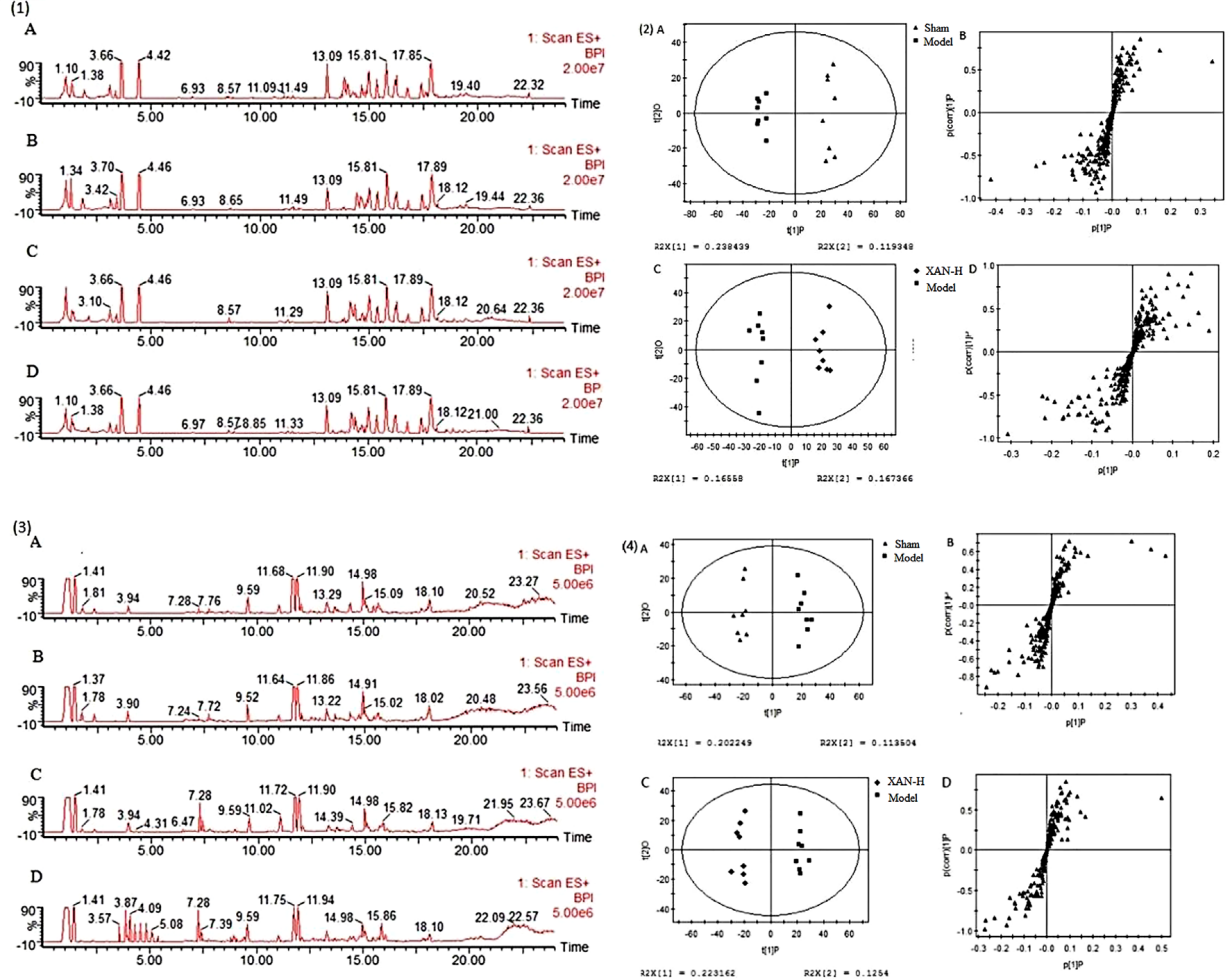
Typical base peak intensity (BPI) chromatograms of plasma (1) and brain (2) samples in positive ion mode from sham **(A)**, model **(B)**, positive drug **(C)**, and high dose of xanthoceraside administrated **(D)** groups. OPLS score plots (A, sham group and model group; C, Xan-L group and model group) and S-plot (B, sham group and model group; D, Xan-L group and model group) based on the plasma (3) and brain (4) metabolic profiling by SIMCA-P 11.0 (n = 8, except in positive drug group, n = 6).

Typical base peak intensity (BPI) chromatograms of plasma and brain were shown in **Figure 3** (1) and (3) that was difficult to directly visualize the differences of chromatograms between samples. The subtle changes among these complex data could be found using a pattern recognition approach. The OPLS score plots (**Figures 3(2)-A, C, (4)-A, C**) showed obvious separation of the sham group with model group and the model group with XAN-H group, which suggested that biochemical perturbation in plasma and brain samples remarkably happened within different groups.

**Figure 4:**
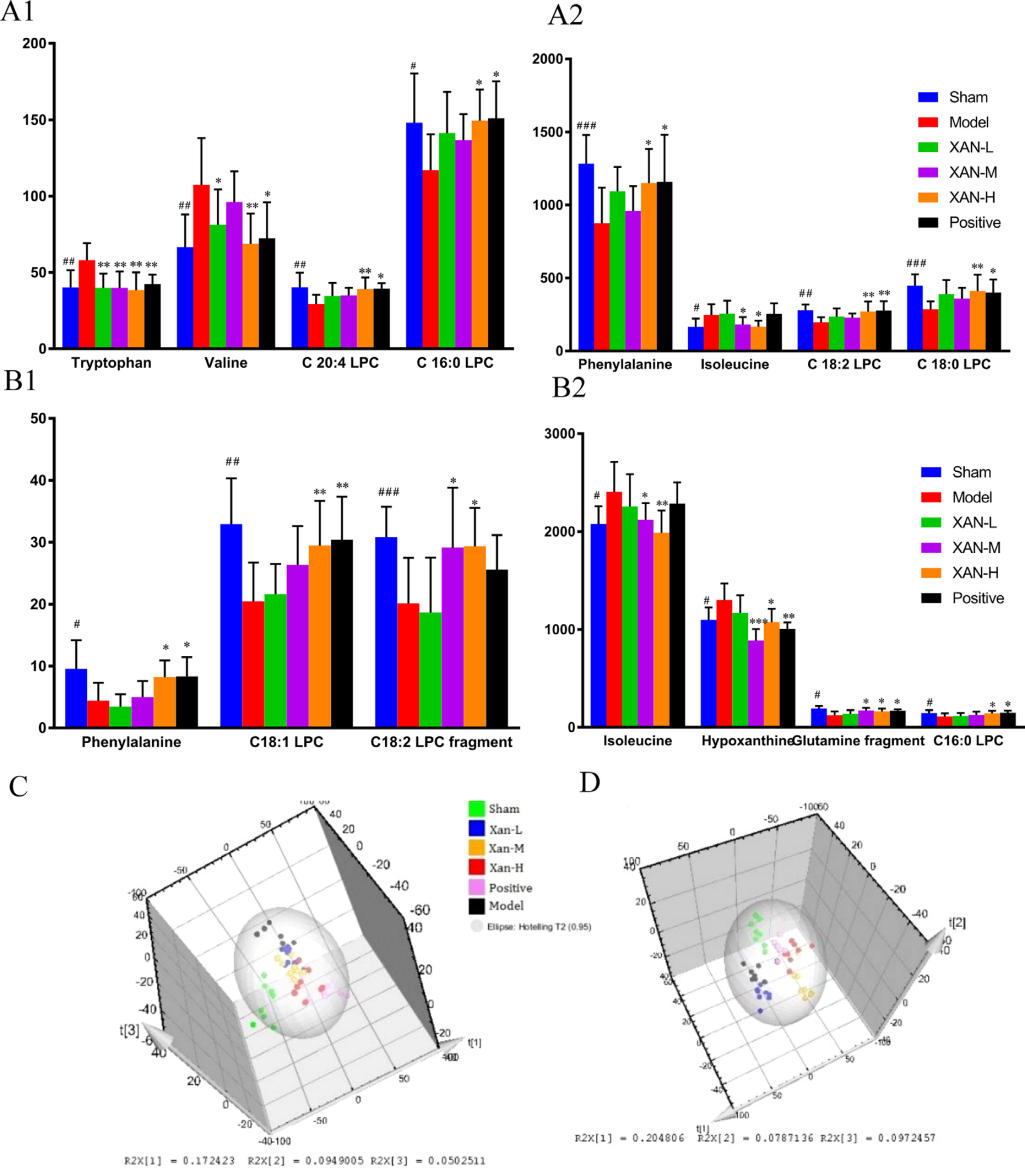
Potential biomarkers detected by UHPLC/MS and their intensities in sham, model, donepezil, and XAN groups. **(A)**. Plasma sample. **(A1**, **A2)** The change metabolites of plasma sample. **(B)**. Brain sample. **(B1**, **B2)** The change metabolites of brain sample. PLS-DA score plots of six groups based on the plasma **(C)** and brain **(D)** metabolic profiling by SIMCA-P 11.0 (n = 8, except in positive drug group, n = 6, ^#^ *p* < 0.05, ^##^ *p* < 0.01 *vs.* sham group; *p < 0.05, **p < 0.01 *vs.* model group; the results are expressed as mean ± SD). ***p < 0.001, ^###^ *p* < 0.001.

Corresponding S-plot (**Figures 3(2)-B, D, (4)-B, D**) revealed the metabolites accountable for the separation. The eight and seven significant variables were detected in plasma and brain samples, respectively, by using ANOVA. The results were summarized in **Figures 4A, B**. Valine and tryptophan were validated by standards, while others were validated by MS/MS analysis and database.

To determine whether the metabolic profiling in plasma and brain tissue differed among six groups, PLS-DA was applied to evaluated separation (**Figures 4 C, D**). In score scatter 3D plot, the sham operation group and model group were obviously separated, revealing the successful establishment of AD animal model. Compared with the model group, the drug administration groups were closer to the sham operation group, indicating the XAN administration and positive drug could alleviate the symptoms of AD. And the three dosage groups of XAN represented a dosage-dependent trend.

#### Gut Microbiota Changes of XAN Treatment in AD Rats

**Figure 5:**
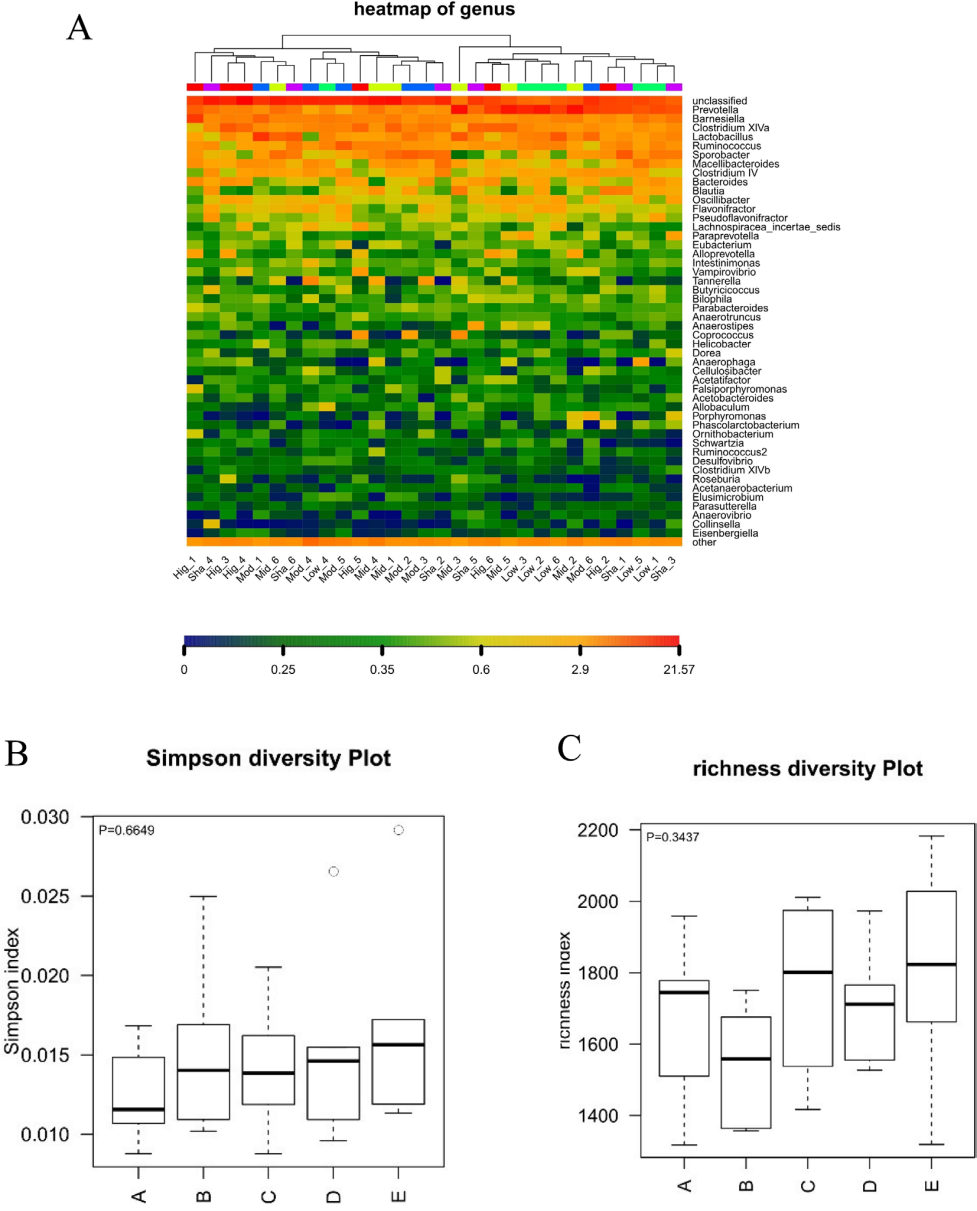
Analysis of alpha diversity in the gut microbiota. A: XAN high-dose groups. B: XAN middle-dose groups. C: XAN low-dose groups. D: AD model groups. E: Sham-operated groups (n = 6/group). **(A)** Species richness hot map in genus. **(B)** Richness of the gut microbiota. **(C)** Simpson index of the gut microbiota.

**Figure 6:**
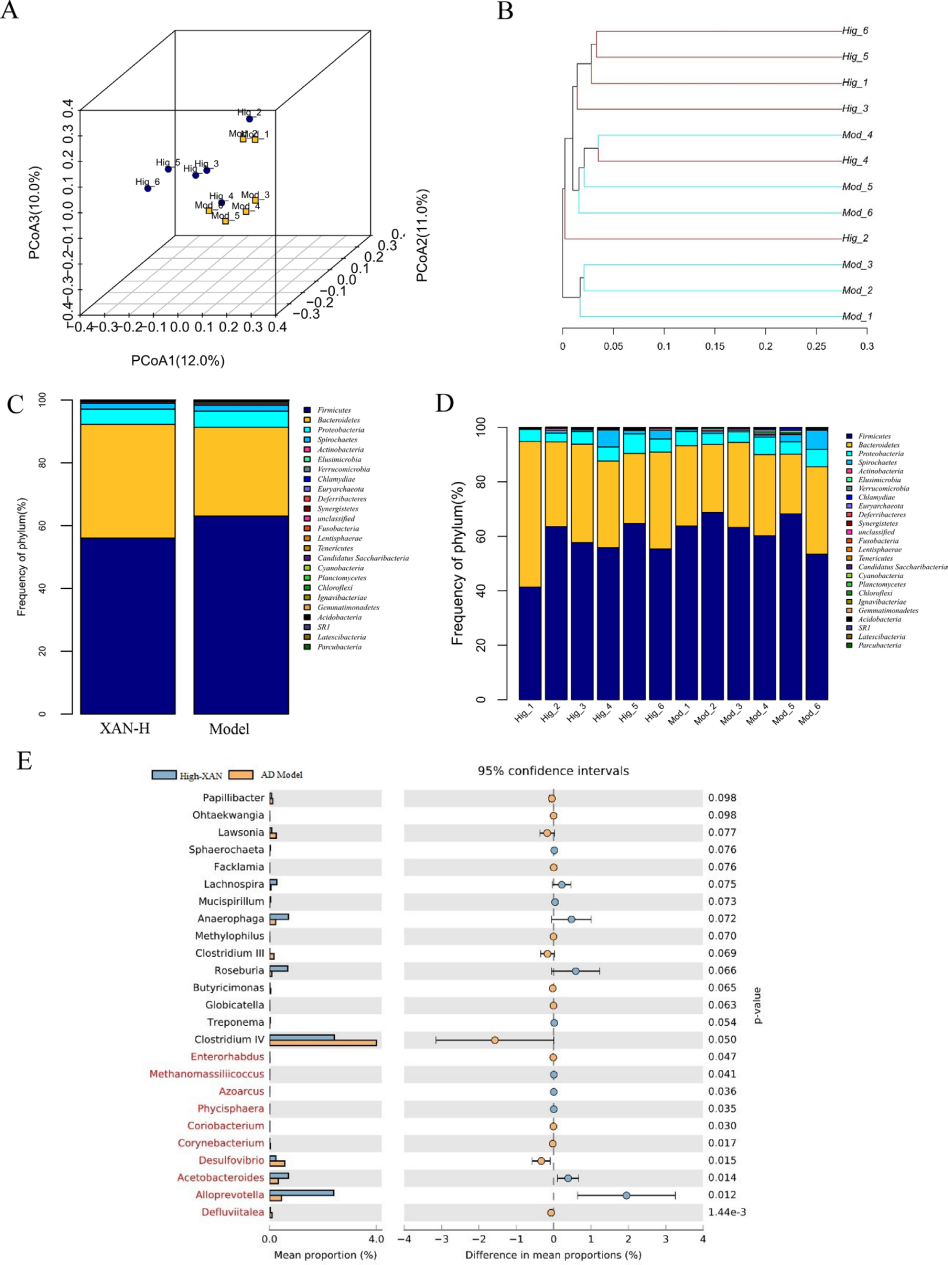
**(A)** PCoA box plots of OTUs between the AD model and XAN high-dose groups. **(B)** Hierarchical clustering analysis of OTUs by unweighted pair group method with arithmetic in the XAN high-dose and model groups. The distance tree was constructed at a distance of 0.1. **(C)** Relative abundance (%) at the phylum level in the XAN high-dose and model groups. **(D)** Differences in relative abundance between XAN high-dose and model groups. **(E)** Gut microbiota changes of XAN treatment in AD rats. Data are expressed as means ± SEM. Analysis shows the relative abundance of microbial genus based on Welch’s test (p ﹤ 0.05). The colored circles represent 95% confidence intervals calculated using Welch’s inverted method. A: XAN high-dose groups. D: AD model groups (n = 6/group).

In present study, the high dose of XAN (XAN-H 0.224 mg/kg) showed a significant therapeutic action for AD in all behavior studies. Therefore, we focus on the change of the model group and the high dose of XAN group. Fecal profiles were analyzed by the 16S rRNA gene sequencing-based method. Alpha diversity analysis suggested that there were no significant differences between XAN treatment and model groups (**Figures 5A, B, C**). Principal coordinate analysis (PCoA) from sequences at the OTU level with >97% similarity was performed by comparing bacterial community patterns. The PCoA score plot revealed that the community composition of the AD model group was far from that of the XAN high-dose group (**Figure 6A**). Moreover, hierarchical clustering analysis using the unweighted pair group method with arithmetic mean also showed a clear separation of XAN high-dose group from AD model group (**Figure 6B**). In the total of bacterial phyla analysis (**Figures 6C, D**), compared with model group, the ratio of *Firmicutes/Bacteroidetes* was decreased in XAN high-dose groups. In addition, 11 genera showed statistically significant differences between XAN high-dose and model groups (**Figure 6E**), in which the relative abundances of *Clostridium IV, Enterorhabdus, Coriobacterium, Corynebacterium, Desulfovibrio*, and *Defluviitalea* in the XAN high-dose group decrease significantly (*p* <0.05), and those of *Methanomassiliicoccus, Azoarcus, Phycisphaera, Acetobacteroides*, and *Alloprevotella* increase significantly (P < 0.05).

#### Potential Relations Between Host Metabolites and Gut Microbiota

**Figure 7:**
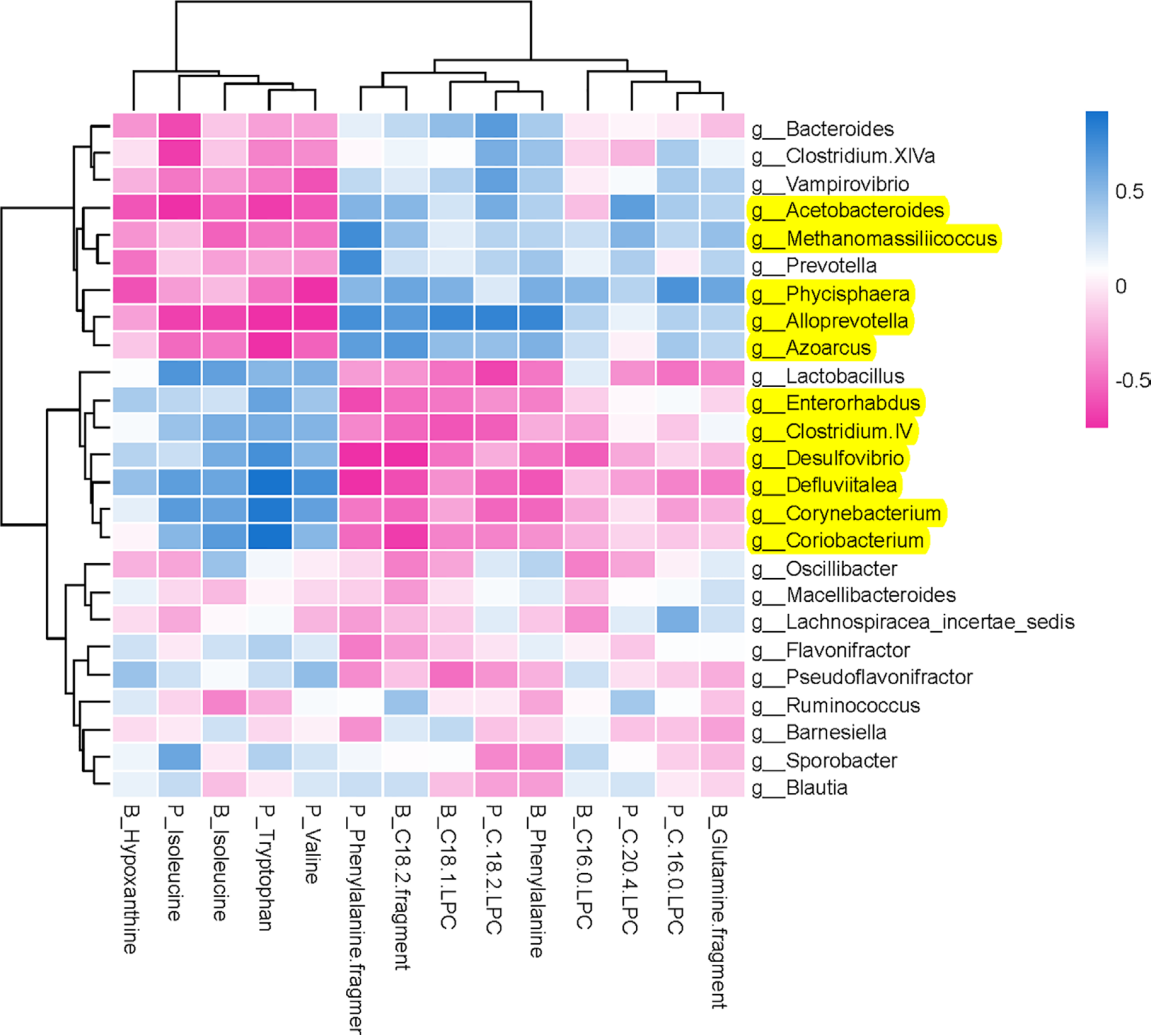
Potential relations between host metabolites and gut microbiota. The intensity of the colors represented the degree of association (blue, positive correlation; red, negative correlation).

To comprehensively analyze the relations between host metabolites and gut microbiota, a correlation matrix was generated by calculating the Spearman’s correlation coefficient (**Figure 7**). In the relationship between host metabolites and gut microbiota, the significant increases of five bacterial strains in XAN high-dose group, including *Methanomassiliicoccus, Azoarcus, Phycisphaera, Acetobacteroides*, and *Alloprevotella*, had negative correlations with five host metabolites, B_Hypoxanthine, P_Isoleucine, B_Isoleucine, P_Tryptophan, and P_Valine, respectively, and positive correlations with nine host metabolites, including P_Phenylalanine fragment, P_C 20:4 LPC, P_C 16:0 LPC, P_C18:2 LPC, B_C18:1 LPC, B_Phenylalanine, B_Glutanine fragment, B_C16:0, and B_C18:2 fragment, respectively. The significant decreases of six bacterial strains in XAN high-dose group, including *Clostridium IV, Enterorhabdus, Coriobacterium, Corynebacterium, Desulfovibrio*, and *Defluviitalea*, had negative correlations with nine host metabolites, P_Phenylalanine fragment, P_C 20:4 LPC, P_C 16:0 LPC, P_C18:2 LPC, B_C18:1 LPC, B_Phenylalanine, B_Glutanine fragment, B_C16:0, and B_C18:2 fragment, respectively, and positive correlations with five host metabolites, B_Hypoxanthine, P_Isoleucine, B_Isoleucine, P_Tryptophan, and P_Valine, respectively.

## Discussion

The current study showed that high dose of XAN played outstanding treatment effects on AD animals. As shown in **Figure 1**, compared with the model group, significantly increasing spontaneous alternation behaviors in Y-maze test and reduction of the escape latency behaviors in the MWM had been exhibited in the XAN-treated group. In H&E staining (**Figure 2**), XAN treatment groups had significantly less hippocampal neuronal loss compared with the model group. The similar effects were found in donepezil treatment group, which showed that XAN had well therapeutic effect on AD.

Recently, emerging evidences displayed that GM might play a critical role in the progression of AD (Kumar et al., 2016; Harach et al., 2017; Wu et al., 2017; Zhang et al., 2017). Some traditional Chinese medicines and natural products could treat disease through changing GM metabolites. For example, Gegen Qinlian decoction could ameliorate glucose homeostasis in type 2 diabetes individuals by changing the structures of GM (Xu et al., 2015). Berberine reduced blood lipid and glucose levels in hyperlipidemia individuals through modulating GM (Wang et al., 2017a; Wang et al., 2017b). In our current study, the positive control, donepezil, a characteristic AChE inhibitor, could enter brain tissue through BBB to exert anti-AD effect. It significantly improves the cognitive function of AD patients through inhibiting AChE activity and slowing the breakdown of AChE. Despite significant effect of XAN, it was hardly absorbed by gastrointestinal tract due to its poor solubility and low permeability, let alone cross BBB. As a consequence, XAN might exhibit its bioactivity in gut tract by affecting the GM structures instead of adsorbing into blood.

The changes of GM structures were closely associated with AD in many reports (Harach et al., 2017; Zhang et al., 2017). The similar results were also obtained in our study, in which the ratio of *Firmicutes*/*Bacteroidetes* in AD models normalized after XAN administration was significant higher than that in normal animals. That’s to say XAN could relieve the pathological conditions through regulating the compositions of GM. In Welch’s test, the significant changes of 11 genera were observed between XAN-H group and AD model group. Among them, *Clostridium IV* has been known for its anti-inflammatory activity (Furet et al., 2010), whose increases are closely associated with obesity and type 2 diabetes in mice (Yamaguchi et al., 2016), as well as the risk factors in AD development (Haan, 2006). *Desulfovibrio* could induce the decreased level of short-chain fatty acids (SCFAs) (Sawin et al., 2015), which contribute to protecting the integrity of intestinal endothelium, influence CNS disease pathologic condition (Sampson et al., 2016), and drive the maturation of microglia. It is essential for maintenance of mature microglia (Erny et al., 2015). Because both microglia and inflammation were key factors during AD development (Pimplikar, 2014; Xie et al., 2017), the inhibitory effect of XAN on *Desulfovibrio* and *Clostridium IV* would facilitate the treatment of AD. In addition, the decreased relative abundance of *Corynebacterium* had been previously observed in ASD and depression (Strati et al., 2017; Yu et al., 2017). The decrease of *Enterorhabdus*, which was involved in the metabolism of SCFAs, was also found in ASD (De Theije et al., 2014). It was the first time to report their abundances changes in AD animals.

The FMT experiment further confirmed that XAN could ameliorate learning and memory abilities by influencing GM. In behavior experiments of both Y-maze and WMM tests, the AXF rats exhibited much better learning and memory abilities than those of ASF rats, and equivalent to those of normal animals. Meanwhile, in H&E staining, the hippocampal neuronal loss in both XAN and AXF rats was significantly fewer than that of AD model and ASF rats. The XAN effect in ameliorating Aβ-induced AD symptoms could be contributed by influencing GM structures, instead of absorbing into the blood.

The influences of microbiome on CNS functions can be achieved through microbiota–gut–brain axis. As the core of the microbiota–gut–brain axis, microbiome affects the CNS by neural, endocrine, metabolic, and immunological pathways, respectively (Wang and Kasper, 2014). Among them, the metabolic pathway was of crucial importance and naturally implicit in the microbiome–gut–CNS signaling (Wang and Kasper, 2014). In the endocrinal pathway, intestinal microbes could produce metabolic precursors of hormones and neurotransmitters and thus act as transducers for the gut–endocrine–CNS route (Lyte, 2014). Systematical experiments were carried out herewith to explore the connections between gut and CNS through these two pathways.

In metabolomics study, series of endogenous metabolites were detected, including five amino acids whose metabolisms were closely related with GM as early reported (Sampson et al., 2016; Kawase et al., 2017; Kennedy et al., 2017). Tryptophan was involved in kynurenine pathway and serotonin synthesis. Kynurenine pathway is regulated by GM, and its metabolites could affect CNS function through metabolism pathway (Kennedy et al., 2017). Tryptophan-2,3-dioxygenase (TDO), the rate-limiting enzyme of the kynurenine pathway, was observed in the high expression of AD brain (Yamada et al., 2009) and able to lead to the up-regulation of the kynurenine and the accumulation of quinolinic acid, which were neurotoxic in the development of AD (Guillemin et al., 2005). The abnormal increase of tryptophan in this study could be reversed after XAN administration, implying the effect of XAN on kynurenine pathway.

Phenylalanine participates in catecholamine metabolism for the biosynthesis of dopamine, norepinephrine, and epinephrine (Wissmann et al., 2013). Dopamine facilitates hippocampal acetylcholine release by acting on D1-like receptors that are G protein-coupled and located in hippocampal cholinergic terminals (Moreno et al., 2011). The activation of dopamine D1 receptors enables working memory by enhancing the firing of pyramidal neurons (Mcnamara and Tejero-Cantero, 2014) and protects neurons from synapse dysfunction induced by Aβ (Jurgensen et al., 2011). As a consequence, these neurotransmitters disturbance may be involved in AD development, and the decreased level of phenylalanine had been observed in AD patients (Gonzalez-Dominguez et al., 2015). XAN could partly increase the levels of phenylalanine to ameliorate AD pathology possibly *via* increasing the levels of dopamine *in vivo*.

Isoleucine and valine, branched chain amino acids (BCAA), whose disturbances were observed in AD (Gonzalez-Dominguez et al., 2015; Pan et al., 2016), could pass through the blood–brain barrier catalyzed by L-type amino acid transporter 1 in a competitive mode with long-chain neutral amino acids (LNAA) such as phenylalanine and tryptophan (Choi and Pardridge, 1986). So, the intakes of BCAA will decrease the content of LNAA in brain and reduce the levels of some neurotransmitters in CNS indirectly (Fernstrom, 2005) as observed in our AD animals. But their increases were effectively restored after XAN intakes accompany with the increases of phenylalanine level.

Glutamine is the precursor of the neurotransmitter amino acids including glutamate, aspartate, and γ-amino butyric acid (GABA) (Albrecht et al., 2010), whose metabolism disturbance was associated with GM (Kawase et al., 2017). Previous research suggested that neurotransmitter amino acids, such as higher levels of GABA and glutamate, could be reversed scopolamine-induced cognitive impairments in AD mice (Brouillette et al., 2007). However, lower abundance of glutamine in brain would lead to the decrease of glutamate and GABA, which will finally result in cognitive obstacles (Danysz and Parsons, 2003; Ellis et al., 2015) as observed in current study. After XAN administration, the increased glutamine level indicated its positive effect on AD symptoms by moderating in glutamine metabolic pathway.

Besides amino acids, LPCs, the important biomarkers in AD pathological conditions (Chalbot et al., 2009) were also detected. LPCs are associated with the integrity of neuronal membrane structure and the metabolite of phosphatidylcholine (PC), which participate in the synthesis of the neurotransmitter acetylcholine (Gonzálezdomínguez et al., 2014). In AD pathological condition, the increased lysophospholipid acyltransferase activity and decreased phospholipase A_2_ activity would decrease LPC level (Ross et al., 1998), further lead to the rapid production and accumulation of free fatty acids and lipid peroxides, eventually result in CNS inflammation, oxidative stress, and neurodegenerative diseases (Farooqui et al., 2000). The metabolisms of LPCs will be influenced by GM (Li et al., 2017). Especially *Firmicutes*, it could remarkably alter the plasma level of LPC 18:1. The lower levels of LPCs in brain as observed in our current data and previous report (Chan et al., 2012) will eventually lead to the impaired lecithin metabolism that result in lesions to the cell and CNS disease because of its high lipid content. The disorder of lipid metabolism in the brain and plasma is one of the factors leading to AD, while XAN could partly restore the LPCs’ level.

The metabolism of hypoxanthine was also associated with the GM (Hong et al., 2010; Yu et al., 2017). Hypoxanthine is an important intermediate for the process of purine metabolism, whose concentration is associated with neurodegenerative diseases (Kaddurah-Daouk et al., 2013). Previous studies suggest that purine metabolites might be involved in the pathophysiology of AD (Xiang et al., 2015), which was also proved in current models. In addition, hypoxanthine could enhance acetylcholinesterase activity in the hippocampus (Wamser et al., 2013), which decreases acetylcholine levels and is closely related to the pathophysiology of AD. Moreover, hypoxanthine has also been reported to induce oxidative stress in rat striatum, which is vital to the pathogenesis of AD and will impair brain memory of mice (Bavaresco et al., 2007). Thus, the abnormal concentration of brain hypoxanthine could be one of the factors leading to AD, and XAN may improve the metabolism of hypoxanthine by decreasing its concentration in brain. This alteration maybe attributed with AChE and oxidative stress, stimulated by Aβ aggregation.

Furthermore, the inherent relationship between host metabolites and GM was analyzed employing Spearman’s correlation analysis (**Figure 7**). It was confirmed that the increases of five bacterial strains (*Methanomassiliicoccus, Azoarcus, Phycisphaera, Acetobacteroides*, and *Alloprevotella*) and decreases of six bacterial strains (*Clostridium IV, Enterorhabdus, Coriobacterium, Corynebacterium, Desulfovibrio*, and *Defluviitalea*) were closely correlated with the changes of endougous metabolites. They could increase the levels of nine host metabolites (P_Phenylalanine fragment, P_C 20:4 LPC, P_C 16:0 LPC, P_C18:2 LPC, B_C18:1 LPC, B_Phenylalanine, B_Glutanine fragment, B_C16:0, and B_C18:2 fragment) and decrease of another five host metabolites (B_Hypoxanthine, P_Isoleucine, B_Isoleucine, P_Tryptophan, and P_Valine), respectively.

**Figure 8:**
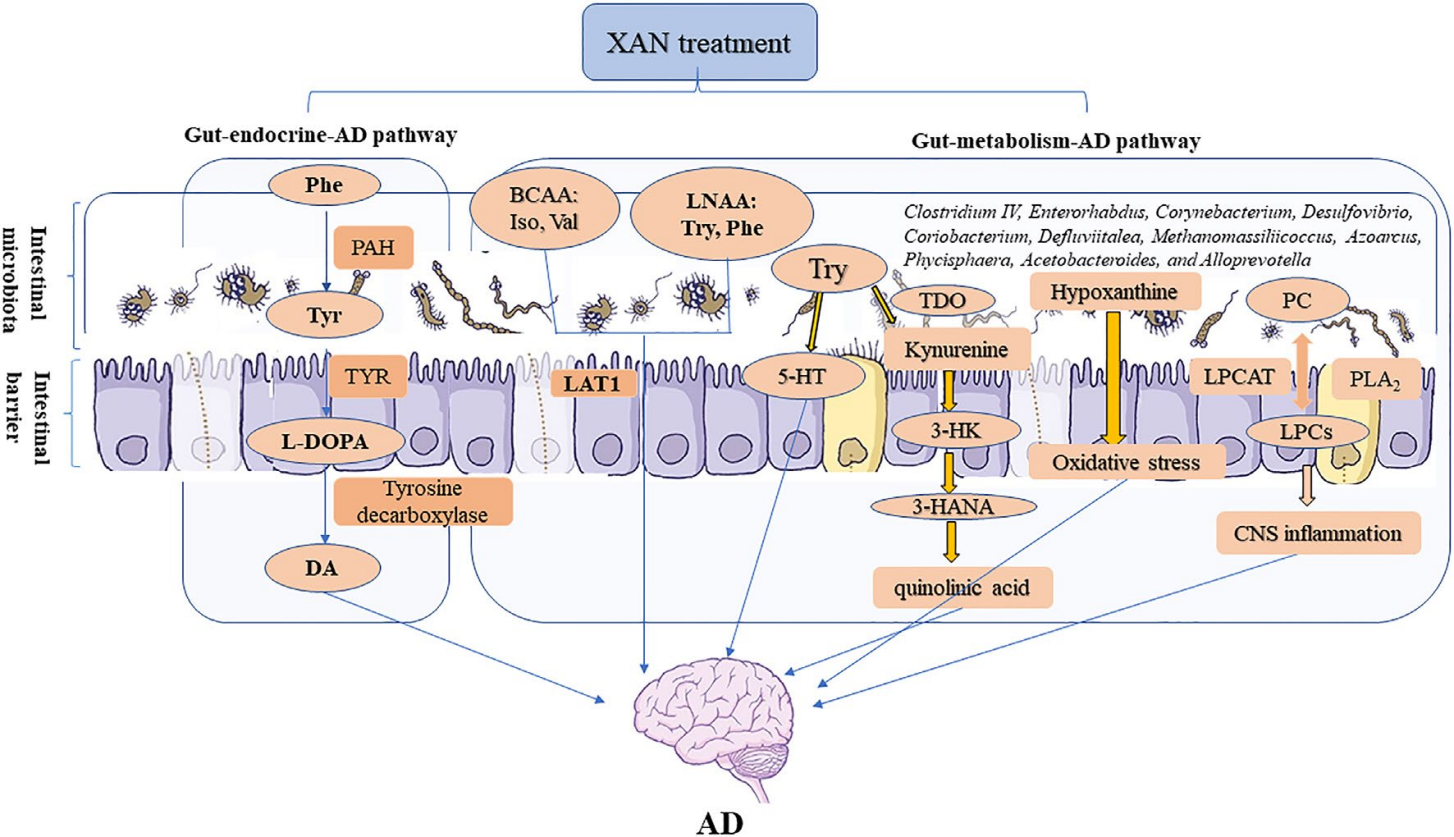
Microbiome–gut–brain axis in relation to AD. In our studies, a serial experiments and analysis were carried out to explore the connections between gut and CNS through metabolic and endocrine pathways. XAN could exert its therapeutic effect by affecting the amino acids metabolism, inflammation, oxidative stress, and purine metabolism *via* gut–metabolic–AD pathway. Simultaneously, XAN could also result in the significant changes of phenylalanine metabolism and finally affect the levels of dopamine and other endogenous substances, which were established as gut–endocrine–AD signaling pathway.

Therefore, the correlation analysis between 16S rRNA gene sequencing and metabolomics study revealed that XAN could modulate the structures of gut microbiota in AD rats, which were consistent with previous reports (Harach et al., 2017; Kobayashi et al., 2017; Zhang et al., 2017), and also significantly correlated with endogenous metabolites ultimately improving AD symptoms. The changes of GM caused by XAN might result in the significant changes of amino acids and LPCs and hypoxanthine, which could be defined as gut–metabolic–AD pathway. Meanwhile, the significant changes of phenylalanine metabolism could be described as gut–endocrine–AD pathway. These two pathways were comprehensively summarized in **Figure 8**.

It must be pointed that many studies had showed the importance of immunology to AD (Wu et al., 2017) and some biomarkers such as dihydrosphingosine, phytosphingosine, and inosine are related to immunological pathways in AD pathology. In addition, SCFAs, produced by GM, also play an important role during AD treatment. Therefore, further studies on neural and immunological pathways in plasma, brain, as well as fecal samples are now under investigations in our group and will be reported later.

## Ethics Statement

All the animal studies were performed in strict accordance with the guidelines of Chinese Society of Laboratory Animal Sciences to minimize the suffering of the animals throughout the study.

## Author Contributions

Conceived and designed the experiments: DM, XL. Performed the experiments: DM, XL, HZ, JT. Analyzed the data: DM, XL, HZ, JT. Contributed reagents/materials/analysis tools: DM, XL. Wrote the manuscript: DM, XL, HZ, HX. All the authors reviewed the manuscript.

## Funding

This work was supported by the National Natural Science Foundation of China (Grant No. 81573694), the Program for Innovative Research Team of the Ministry of Education and Program for Liaoning Innovative Research Team in University, and the program for Innovative team of Liaoning province (Grant No.LT2015027) and the Liaoning BaiQianWan Talents Program.

## Conflict of Interest Statement

The authors declare that the research was conducted in the absence of any commercial or financial relationships that could be construed as a potential conflict of interest.

## Abbreviations

Phe, phenylalanine; Tyr, tyrosine; PAH, phenylalanine-4-hydroxylase; TYR, tyrosinase; L-DOPA, L-dopamine; DA, dopamine; BCAA, branched chain amino acids; LNAA, long-chain neutral amino acids; Iso, isoleucine; Val, valine; Try, tryptophan; Phe, phenylalanine; LAT1, L-type amino acid transporter 1; 5-HT, serotonin; TDO, tryptophan-2,3-dioxygenase; 3-HK, 3-hydroxykynurenine; 3-HANA, 3-hydroxyanthranilic acid; PC, phosphatidylcholine; LPCs, lysophosphatidylcholine; PLA2, phospholipase A2; LPCAT, lysophospholipid acyltransferase.

## Supplementary Material

Figure S1
**(A)** Chemical structure of xanthoceraside. **(B)** Experiment design and drug administration. **(C)** Fecal microbiota transplantation experiment design.

Figure S2 The HPLC chromatogram of xanthoceraside.
