# Supplementary figures and images for: Xanthoceraside Could Ameliorate Alzheimer’s Disease Symptoms of Rats by Affecting the Gut Microbiota Composition and Modulating the Endogenous Metabolite Levels
Source: Front Pharmacol. 2019 Sep 13;10:1035. doi: 10.3389/fphar.2019.01035 (PMC6753234; doi:10.3389/fphar.2019.01035)

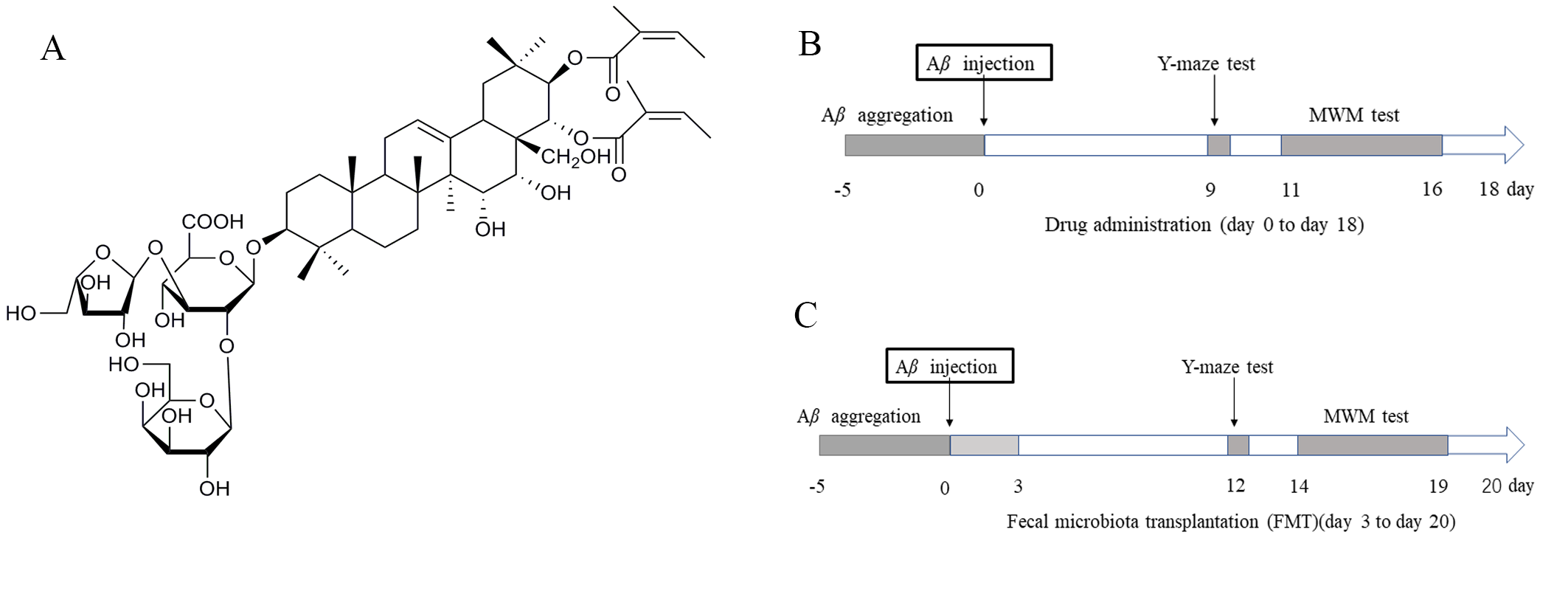

Supplement: Figure S1 — (A) Chemical structure of xanthoceraside. (B) Experiment design and drug administration. (C) Fecal microbiota transplantation experiment design. [file Image_1.tif]

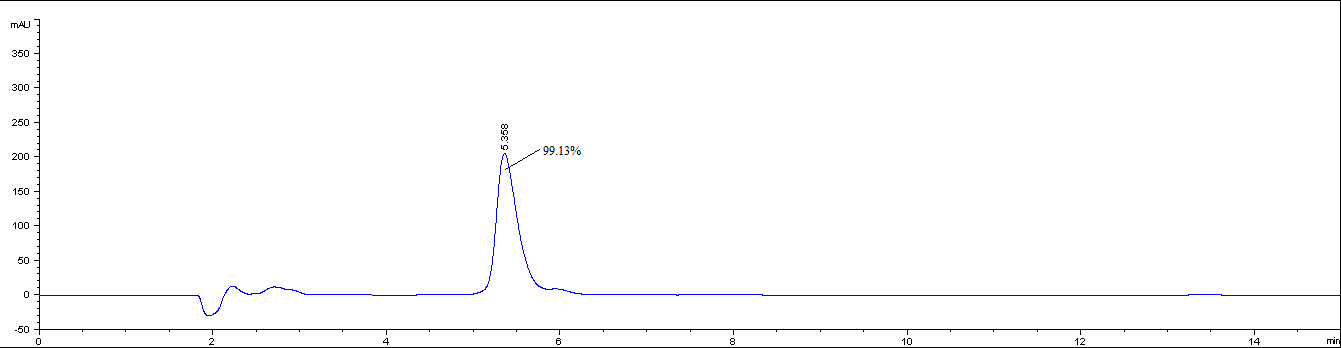

Supplement: Figure S2 — The HPLC chromatogram of xanthoceraside. [file Image_2.tif]
